# Microleakage in Class II composite restorations with margins below the CEJ: In vitro evaluation of different restorative techniques

**DOI:** 10.4317/medoral.18344

**Published:** 2013-05-31

**Authors:** Claudio Poggio, Marco Chiesa, Andrea Scribante, Jenia Mekler, Marco Colombo

**Affiliations:** 1 Department of Operative Dentistry, University of Pavia, Policlinico “San Matteo”; 2 Department of Orthodontics, University of Pavia, Policlinico “San Matteo”

## Abstract

Objectives: The purpose of this in vitro study was to evaluate the microleakage in “deep” Class II composite restorations with gingival cavosurface margin below the CEJ (cemento-enamel junction) and restored with different techniques. Study Design: Fifty human teeth were used. In each tooth two standardized Class II slot cavities (on mesial and on distal surfaces) were prepared: the buccolingual extension of the cavities was 4 mm; the gingival wall was located in dentin/cementum (2 mm beyond the CEJ). The prepared teeth were randomly assigned to 5 experimental groups (of 10 specimens and 20 cavities each) and restored. Group 1: Filtek TM Supreme XTE Flowable (3MESPE) + Universal Filtek Supreme XTE (3MESPE), Group 2: GrandioSO Heavy Flow (Voco) + GrandioSo (Voco), Group 3: SDR™ (Dentsply Caulk) + Esthet-X® HD (Dentsply Caulk), Group 4: SonicFill (Kerr), Group 5: Grandio (Voco). After thermocycling, the specimens were immersed in a 0.5% basic fuchsine dye solution and incubated at 37°C for 24 hours. The teeth were subsequently sectioned mesiodistally. All specimens were examined at 25× in a stereomicroscope and standardized digital images were obtained. Dye penetration was measured from gingival margins. Results: The results demonstrated no significant leakage differences between Group 4 and Group 5, that both showed significantly higher frequency distribution of Score 0. Group 2 and Group 3 showed a significant prevalence of Score 1, whereas Group 1 showed significantly higher frequency of Score 2. Conclusions: None of the restorative techniques tested completely eliminated microleakage dye penetration in dentin margins; marginal adaptation in Class II composite restorations with gingival wall below the CEJ varied in both substrates and from different restorative techniques used.

** Key words:**Microleakage, Class II composite restorations, CEJ.

## Introduction

Direct Class II composite restorations can be placed at an acceptable standard if the cervical margin is in sound enamel; when the adhesive restorations are located below the CEJ (cemento-enamel junction) and cervical lesions have no enamel the quality of the marginal integrity is questionable (1). Below the CEJ the bond with dentin is weaker: the polymerization shrinkage can result in gap formation between composite resin and the cavity walls. Marginal gap formation contributes to micro leakage permitting the passage of oral fluids and bacteria from the oral cavity and become a source of postoperative sensitivity, pulpal inflammation and recurrent caries (2-4). To reduce these effects have been suggested, as a better option to the conventional resin technique, the Class II open-sandwich restorations: glass-ionomer cement (GIC) or resin-modified glass-ionomer cement (RMGIC) is placed between the dentin cervical margins and occlusal composite restoration. (5,6). GICs and RMGICs have been shown to be less able to seal margins, can dissolve over time in the oral environment (7-9). Recently flowable resin composites (FRC), with lower filler content and far lower viscosity, have been recommended as liners at CEJ margins of the proximal box of Class II composite restorations to improving marginal integrity and to resulting less micro leakage and post-operative sensitivity: (4,10) a layer of flowable materials at the gingival floor (in cementum margins) of Class II composite restorations get better the marginal seal of a restoration and is an ideal choice for use in a open-sandwich technique (11-13). One approach to improving the marginal seal and reducing micro leakage is to use a flowable composite resin under highly filled composite restorations: however several studies do not show improved performance (10,14). The most recent attempt to reduce micro leakage uses new resin monomers with novel chemistries (low polimerization shrinkage) to compensate shrikage stress. SDR™(Dentsply Caulk) is designed to reduce micro leakage by increasing flow with a unique chemistry that slows the rate of polimerization to reduce shrinkage stress This composite resin is used as a dentin replacement material and polymerized in 4-mm increments (15). Sonic Fill is a single-step composite system that doesn’t require an additional capping layer. Sonic Fill System combines the advantages of a flowable composite with a universal composite. Sonic Fill System is comprised of a KaVo hand piece that enables sonic activation of a specially designed and conveniently delivered composite from Kerr. Sonic Fill’s activation significantly reduces the composite’s viscosity to rapidly fill the cavity. The purpose of this in vitro study was to evaluate the micro leakage in “deep” Class II composite restorations with gingival cavosurface margin below the CEJ and restored with different techniques. The null hypothesis of the study was that there is no significant difference in micro leakage of the different evaluated restorative techniques evaluated.

## Material and Methods

-Specimen preparation

Fifty caries-free vital human teeth freshly extracted for periodontal or orthodontic reasons were used in this study. The teeth were cleaned with dental scalers, polished with pumice and stored in a 0.25% mixture of sodium azide in Ringer solution until the date of use. In each tooth two standardized Class II slot cavities (on mesial and on distal surfaces) were prepared with a round-nosed no.245 carbide bur (Dentsply/Caulk, Milford, DE, USA) at high-speed with air/water spray (16). The buccolingual extension of the cavities was 4 mm; the gingival wall was located in dentin/cementum (2 mm below the cementum-enamel junction/CEJ); the internal angles were rounded and cavosurface margins were finished with gingival margin trimmers (17). The prepared teeth were randomly assigned to 5 experimental groups (of 10 specimens and 20 cavities each) and were mounted in a jig featuring artificial training teeth that served as adjacent teeth. A contoured matrix band was placed around the teeth for restorative procedures. The same trained operator prepared all the cavities.

-Restorative procedure

Group 1. The cavities were etched with 37% phosphoric acid for 30 seconds (Total Etch; Ivoclar Vivadent AG, Schaan, Liechtenstein) and bonded with Adper Scotchbond 1 XT (3M ESPE, St.Paul, MN, USA). A layer (approximately 1 mm in thickness) of a flowable material (Filtek TM Supreme XTE Flowable/3MESPE, St.Paul, MN, USA) was placed (by a periodontal probe) to cover the entire gingival floor of the cavity. The cavities were then restored with a “nanofilled” composite (Universal Filtek Supreme XTE/3MESPE, St.Paul, MN, USA), using a horizontal incremental technique with 3 horizontals increments (2 mm thick) from the cervical to the occlusal surface.

Group 2. The cavities were etched with 37% phosphoric acid for 30 seconds (Total Etch; Ivoclar Vivadent AG, Schaan, Liechtenstein) and bonded with Adper Scotchbond 1 XT (3M ESPE, St.Paul, MN, USA). A layer (approximately 1 mm in thickness) of a flowable material (GrandioSo Heavy Flow/Voco GmbH, Cuxhaven, Germany) was placed (by a periodontal probe) to cover the entire gingival floor of the cavity. The cavities were then restored with a “nanoybrid” composite (GrandioSo/Voco GmbH, Cuxhaven, Germany), using a horizontal incremental technique with 3 horizontals increments (2mm thick) from the cervical to the occlusal surface.

Group 3. The cavities were etched with 37% phosphoric acid for 30 seconds (Total Etch; Ivoclar Vivadent AG, Schaan, Liechtenstein) and bonded with Adper Scotchbond 1 XT (3M ESPE, St.Paul, MN, USA). The flowable material (SDR™/Dentsply Caulk, Mildford, DE, USA) was placed in a 4 mm bulk increments and light cured for 20 seconds. An occlusal layer of Esthet-X® HD (Dentsply Caulk, Mildford, DE, USA) composite was added on top to build the final anatomy of the teeth and to complete the restoration.

Group 4. The cavities were etched with 37% phosphoric acid for 30 seconds (Total Etch; Ivoclar Vivadent AG, Schaan, Liechtenstein) and bonded with Adper Scotchbond 1 XT (3M ESPE, St.Paul, MN, USA). The flowable material (Sonic Fill/Kerr, West Collins, Orange, CA, USA) was placed with the Sonic Fill Hand piece (sonically activated delivery) in a 4 mm bulk increments and light cured for 20 seconds. Sonic Fill is a single-step composite system that doesn’t require an additional capping layer.

Group 5. (control). The cavities were etched with 37% phosphoric acid for 30 seconds (Total Etch; Ivoclar Vivadent AG, Schaan, Liechtenstein) and bonded with Adper Scotchbond 1 XT (3M ESPE, St.Paul, MN, USA). The cavities were totally restored with a “nanohybrid” composite (Grandio/Voco GmbH,Cuxhaven, Germany), using a horizontal incremental technique with 3 increments from the cervical to the occlusal surface (each increment being 2 mm).

Each layer or increment was cured for 20 seconds from the occlusal surface with a LED curing light in soft start-polymerization mode (Celalux 2 High-Power LED curing-light, Voco GmbH, Cuxhaven, Germany) for 20 seconds at a light intensity of 1000 mW/cm2 according to manufacturers’ instructions. Then the metallic matrix was removed and the restorations were light cured for 20 seconds from the buccal and lingual surfaces and the surface was finished and polished with finishing/polishing disks (Sof-Lex Pop-On, 3M ESPE, St. Paul, MN, USA) in decreasing granulation. All teeth were coated with two layers of nail varnish up to 1 mm from the restorations margins, while the apical part was sealed with wax. The restored teeth were then subjected to artificial aging by thermocycling. All specimens were immersed alternately in water baths at 5 and 60°C for 1500 cycles, with at dwell time of 60 seconds in each bath and a transfer time of 15 seconds. After thermocycling, the specimens were immersed in a 0.5% basic fuchsine dye solution and incubated at 37°C for 24 hours. The teeth were subsequently rinsed for 10 minutes under running water to remove external dye, dried and sectioned mesiodistally through the centre of the restorations with a low-speed water-cooled diamond cutter.

-Micro leakage analysis

All specimens were examined at 25X in a stereo microscope (Inspective 4Geek, Serravalle, RSM) and standardized digital images were obtained. Two observers scored each section blindly; consensus was forced if disagreements occurred. Dye penetration was measured from gingival margins. An independent examiner did scoring; another trained examiner confirmed observations. The cervical marginal micro leakage was recorded based on the following criteria (18): score 0 = no dye penetration, score 1 = dye penetration limited to enamel, score 2 = dye penetration beyond the dentin-enamel junction but limited to 2/3rds of the cervical wall length, score 3 = dye penetration beyond 2/3rds of the cervical wall length but not to the pulpal wall, score 4 = dye penetration to the pulpal wall.

-Statistical analysis

The results of micro leakage scores were subjected to statistical analysis using “Stata 7.0” computer software (Stata Corp., Station College, TX). As the data are on an ordinal scale, a Kruskal-Wallis test was used to assess differences among the different groups. Mann-Whitney U test was used as post hoc to investigate pairwise differences. Significance was predetermined for P<0.05.

## Results

**Figure 1:**
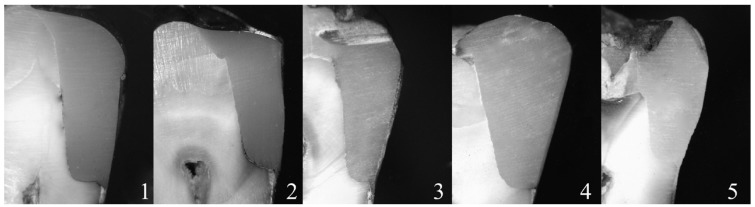
Representative stereomicroscopic photograph of the different Groups (original magnification 25×).

**Figure 2:**
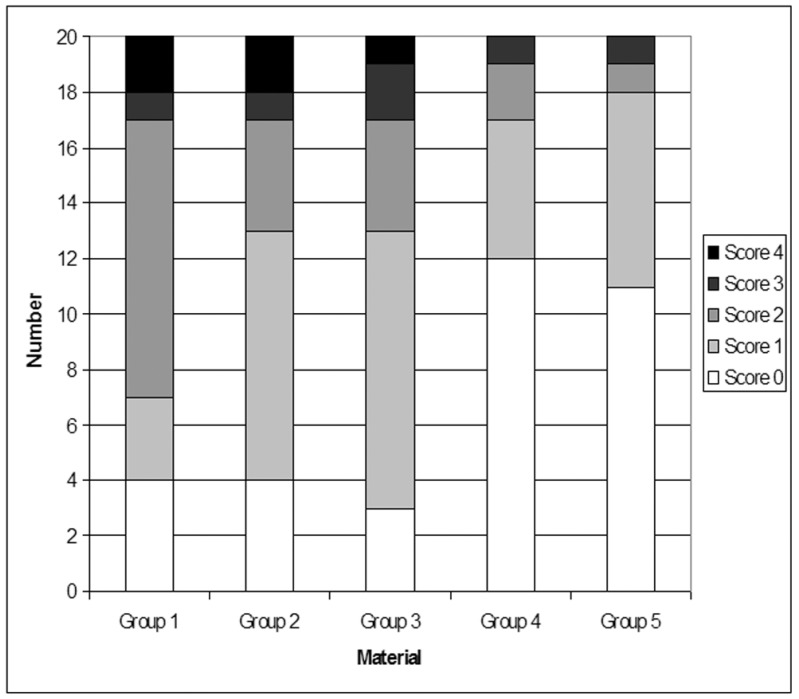
Distribution of ARI scores of the different groups.

Representative stereo microscopic photograph of micro leakage in Groups 1 to 5 are showed in figure 1. Micro leakage scores for the dentin margins are presented in  Table 1 and illustrated in figure 2. The results demonstrated no significant leakage differences between Group 4 and Group 5, that both showed significantly higher frequency distribution of Score 0. Group 2 and Group 3 showed a significant prevalence of Score 1, whereas Group 1 showed significantly higher frequency of Score 2 (Fig 2).

**Table 1:**
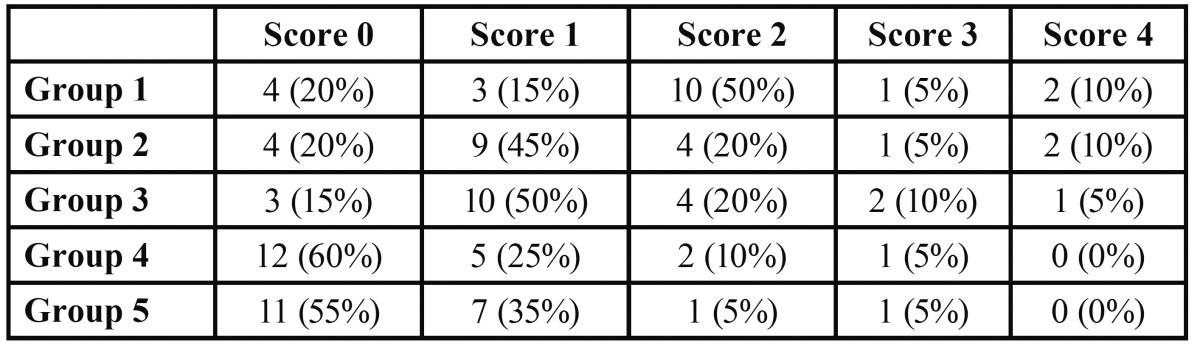
Frequency distributions of microleakage scores (percentages) on dentin margins among the different groups tested.

## Discussion

The null-hypothesis of the study has been rejected. In the present investigation none of the adhesive systems tested completely eliminated micro leakage in dentin margins of the cavity. This is in agreement with previous studies that evaluated micro leakage of restoration at dentin interface (12,19-21).

Significant prevalence of Score 0 (no dye penetration) was reported both for Groups 4 (Sonic Fill) and 5 (Grandio), thus indicating that both composites showed the lowest micro leakage values when compared with other groups tested. Higher micro leakage scores were recorded for Groups 2 (GrandioSo Heavy Flow+GrandioSo) and 3 (SDR+ Esthet-X HD), that both showed significant prevalence of Score 1 (dye penetration limited to enamel). The highest dye penetration values were reported for Group 1 (Filtek TM Supreme XTE Flowable+Universal Filtek Supreme XTE) that showed a significant prevalence of Score 2 (dye penetration beyond the den tin-enamel junction but limited to 2/3rds of the cervical wall length). None of the Groups tested in the present investigation showed a significant prevalence of Score 3 and Score 4, thus indicating that the median of the scores reported was not correlated with a dye penetration beyond the den tin-enamel junction over 2/3rds of the cervical wall length or over pulpal wall (18).

Micro leakage, due to microscopic openings between the margins of the composite restoration and the tooth, is considered a major cause of restoration failure (22). Dye penetration values obtained from in vitro studies are often higher than those obtained in vivo (23). In fact Micro leakage tests have been widely employed to screen the seal efficiency of restorations. Such tests face the challenge of reproducing the oral dynamics in an in vitro assay. Their results tend to present high variability, probably, due to different test methods (24). Micro leakage was chosen in this study because of its long-term report in literature. Furthermore, the test was designed taking into consideration the most frequent choices in test variables, as reported by Raskin, et al. (24) in a systematic literature review.

Micro leakage can result in bacteria penetrating the tooth-restoration space and into dentinal tubules, where secondary decay may occur and bacterial toxins will irritate the pulp. The oral environment (including occlusal forces and temperature variation) and several differences between the physical properties of teeth and restorative materials (including polymerization shrinkage, coefficient of thermal expansion, and modulus of elasticity) can contribute to micro leakage (25). According to previous literature, if poor bond strength exists between the tooth and restorative material, a failure of adhesion may be caused by polymerization shrinkage, and microscopic gaps at the tooth/restoration interface can subsequently form (26,27). Micro leakage, either from small or microscopic openings between the margins of the composite restoration and tooth, was considered a major cause of restoration failure (28,29). The majority of Class II cavities exhibit cavity margins with gingival wall below the CEJ in both dentine and/or cementum (30). Therefore, the cervical margins of restorations will be placed at dentine or cementum surfaces, which may lead to a weaker marginal seal than at the enamel surface (30). This in vitro study examined the micro leakage in “deep” Class II composite restorations with gingival cavosurface margin below the CEJ and restored with different techniques. Within the limitations of this in vitro study, none of the restorative techniques tested completely eliminated micro leakage dye penetration in dentin margins; marginal adaptation in Class II composite restorations with gingival wall below the CEJ varied in both substrates and from different restorative techniques used.
